# Pressurized intra-peritoneal aerosol chemotherapy (PIPAC): increased intraperitoneal pressure does not affect distribution patterns but leads to deeper penetration depth of doxorubicin in a sheep model

**DOI:** 10.1186/s12885-021-07955-w

**Published:** 2021-04-26

**Authors:** Myriam Mimouni, Christophe Richard, Pierre Adenot, Martine Letheule, Anne Tarrade, Olivier Sandra, Michèle Dahirel, Thomas Lilin, Benoit Lecuelle, Valérie Gélin, Julien Cohen, Arnaud Fauconnier, François Vialard, Cyrille Huchon, Pascale Chavatte-Palmer

**Affiliations:** 1 Université Paris-Saclay, UVSQ, INRAE, BREED, 78350 Jouy-en-Josas, France; 2 Ecole Nationale Vétérinaire d’Alfort, BREED, 94700 Maisons-Alfort, France; 3 Department of Gynecology and Obstetrics, 10 rue du Champ Gaillard, CHI Poissy-Saint-Germain, 78300 Poissy, France; 4 Centre de Recherche BioMédicale (CRBM), ENVA, UPE, Maisons-Alfort, France; 5 Medistat, Biostatistics, 10-12 rue de la Conception, 13004 Marseille, France; 6 Department of Pharmacy, 10 rue du Champ Gaillard, CHI Poissy-Saint-Germain, Poissy, France; 7 EA 7285 Clinical Risks and Safety on Women’s Health, University Versailles-Saint-Quentin en Yvelines, 2 avenue de la Bièvre, 78180 Montigny le Bretonneux, France; 8 APHP. Department of Gynecology and Obstetrics, Hôpital Lariboisière, University of Paris, 2, rue Ambroise Paré, 75010 Paris, France

**Keywords:** Sheep, Doxorubicin, PIPAC, Intraperitoneum pressure, Peritoneal carcinomatosis

## Abstract

**Background:**

Pressurized Intra-Peritoneal Aerosol Chemotherapy (PIPAC) is an innovative treatment against peritoneal carcinomatosis. Doxorubicin is a common intra-venous chemotherapy used for peritoneal carcinomatosis and for PIPAC. This study evaluated the impact of increased PIPAC intraperitoneal pressure on the distribution and cell penetration of doxorubicin in a sheep model.

**Methods:**

Doxorubicin was aerosolized using PIPAC into the peritoneal cavity of 6 ewes (pre-alpes breed): *N* = 3 with 12 mmHg intraperitoneal pressure (“group 12”) and N = 3 with 20 mmHg (“group 20”). Samples from peritoneum (*N* = 6), ovarian (*N* = 1), omentum (N = 1) and caecum (N = 1) were collected for each ewe. The number of doxorubicin positive cells was determined using the ratio between doxorubicine fluorescence-positive cell nuclei (DOXO+) over total number of DAPI positive cell nuclei (DAPI+). Penetration depth (μm) was defined as the distance between the luminal surface and the location of the deepest DOXO+ nuclei over the total number of cell nuclei that were stained with DAPI. Penetration depth (μm) was defined as the distance between the luminal surface and the location of the deepest DOXO+ nuclei.

**Results:**

DOXO+ nuclei were identified in 87% of samples. All omental samples, directly localized in front of the nebulizer head, had 100% DOXO+ nuclei whereas very few nuclei were DOXO+ for caecum. Distribution patterns were not different between the two groups but penetration depth in ovary and caecum samples was significantly deeper in group 20.

**Conclusions:**

This study showed that applying a higher intra-peritoneal pressure during PIPAC treatment leads to a deeper penetration of doxorubicin in ovarian and caecum but does not affect distribution patterns.

## Background

Peritoneal carcinomatosis (PC) is a peritoneal metastasis of many cancers, especially ovarian cancer. In France, ovarian cancer affects 4600 women and induces 3200 deaths annually (Institut National du Cancer 2015). The first intention treatment is the association of complete surgery in addition to platinum based-chemotherapy [1]. PC often extends to the whole abdomen, from the diaphragm peritoneum down to the pelvis. The extensive size of the affected zone is the main difficulty for the surgical treatment of ovarian cancer as completeness of the initial surgery is one of the two main prognostic factors. Resistance to chemotherapy is the second most important reason for relapse [2]. Despite optimal treatment, 70% of patients with ovarian cancer relapse within 5 years [3] and 1 in 4 will become platinum-resistant (relapse within 6 months after platinum-containing therapy) [4]. For these patients, therapeutic possibilities become rare and prognosis is poor [5]. Although the recent availability of bevacizumab treatment improved the survival rate of these patients, surgery is rarely feasible and the effects of chemotherapy remain limited. Thus, finding new therapies for these patients remains urgent [6].

In most cases, ovarian cancer is restricted to the peritoneal cavity without distant organic metastasis (stade IIIC in FIGO classification) [7]. This is the ideal target for intra-peritoneal treatment. In 2012, a new method for intra-peritoneal administration of chemotherapy, Pressurized Intra-Peritoneal Aerosol Chemotherapy (PIPAC), was developed, where the chemotherapy is nebulized at body temperature in the intra-peritoneal cavity during laparoscopy [8]. The conversion of liquid chemotherapy into droplets is thought to enable homogeneous peritoneal distribution. Moreover, compared to a simple lavage, drug administration under the pressure used for the laparoscopy was shown to induce a better penetration of drugs in an in vitro model [9]. Finally, the plasmatic uptake of chemotherapeutic drugs is negligible, thus limiting side effects of chemotherapy [10, 11]. The standard intra-abdominal pressure used in the initial published protocol was 12 mmHg [12], which has been applied for clinical use.

So far, clinically, doxorubicin, which is commonly used for the chemotherapy of ovarian cancers, is also used with the PIPAC procedure. It acts through the inhibition of DNA transcription. Three interventions at 4–6 weeks interval each were shown to largely reduce peritoneal carcinomatosis [13, 14]. Furthermore, the patients’ quality of life of being seems to be maintained when treated with PIPAC chemotherapy [15, 16]. These encouraging pioneer data prompt the needs for further evaluation and improvement [17, 18].

In this context, the objective of our study was to compare the penetration and the distribution of doxorubicin administered with PIPAC using two distinct intra-peritoneal pressures (12 and 20 mmHg).

Experiments were carried out in sheep, of similar size and weight to humans, so that the same equipment could be used. None of the large domestic animals spontaneously nor experimentally develop ovarian cancer similar to humans, so a healthy model was used.

## Methods

### Ethical statement

The project was approved by the local ethics committee (N°16 in the French registry of ethical committees) of animal experimentation of the National Veterinary School of Alfort and validated by the French Ministry of Research under registration “APAFIS” number 2016113016134972. Sheep were euthanized under general anaesthesia after PIPAC procedure and before sampling (Directive 2010/63/UE of European Parliament and Council dated September 22nd, 2010). This was performed by a trained team. All precautions were taken to limit anxiety and pain of the animals.

### Experimental plan

Altogether, 10 non-pregnant multiparous ewes were used. The first three animals were used for preliminary tests and development of the model. Thereafter, PIPAC was carried out as follows: (i) one control female with physiological serum, (ii) three females with a capnoperitoneum at 12 mmHg (group 12), and (iii) 3 females with a capnoperitoneum at 20 mmHg (group 20). To avoid a potential “day” effect, procedures for group 12 and 20 were performed alternatively (2–3 procedures/day). Animal characteristics are described in Table 1.

**Table 1:** Characteristics of ewes used for PIPAC experiments

Sheep (procedural order)	Weight (Kg)	Capnoperitoneum (mmHg)	Group
control	52	12	control
1	51	12	1
2	46	20	2
3	54	12	1
4	47	20	2
5	45	12	1
6	51	20	2

### Surgical procedure

All PIPAC procedures were performed in the surgery theatre of the Biomedical research center (CRBM) of the National Veterinary School of Alfort.

### General anaesthesia

The anaesthesia was carried out by a trained team. Animals were fasted for 12–16 h before surgery. After a premedication with ketamine (Imalgen 1000®, Merial, 4 mg/kg IV) and diazepam (Diazepam, TVM, 0.5 mg/kg IV), anaesthesia was maintained with an automated ventilator, using isofluorane (2–2.5%) diluted in a mixture of air and oxygen (50/50). Analgesia was ensured by IV injection of fentanyl (Fentadon®, Eurovet Animal Health, 2 μg/kg IV) per hour. Per-operating supervision focused on respiratory rate, cardiac frequency, oxygen saturation and arterial pressure.

### PIPAC: surgical procedure

The PIPAC was performed according to the safety rules described by Solaß (2013). All precautions were taken to ensure staff safety: every operator wore a surgical blouse, gloves, protection glasses and a high protection breathing mask.

**Fig. 1:**
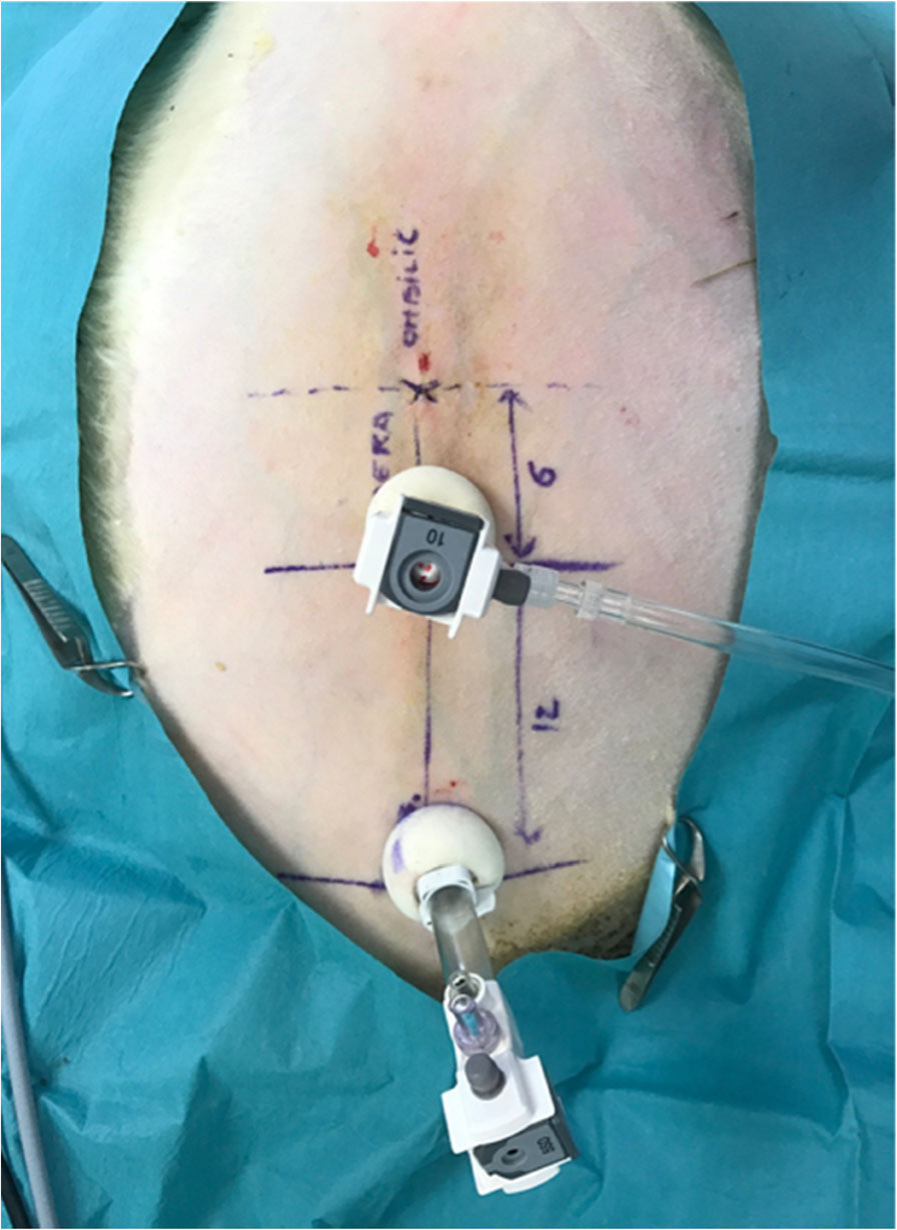
Localization of trocars on sheep’s abdominal wall

**Fig. 2:**
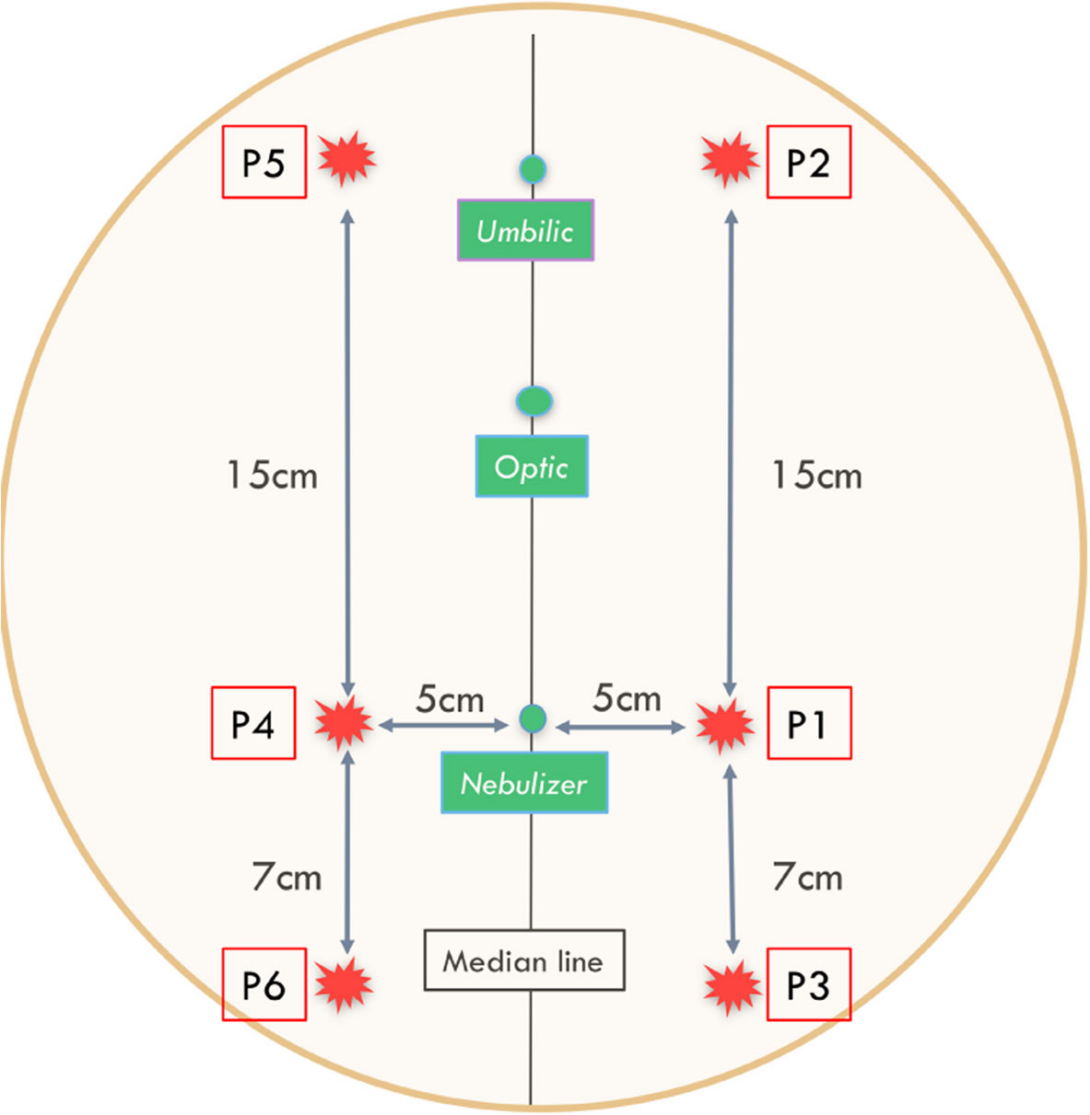
Standardized location of peritoneal samples (P1 to P6) according to distance to nebulizer. Figure 2 Identification of doxorubicin in nuclei. * Nuclei DAPI+: nuclei stained by DAPI. Nuclei DOXO+: nuclei stained by Doxorubicin and DAPI

After clipping the anterior abdominal wall, points were drawn on the skin for trocar localization, 6 cm (laparoscopic camera) and 18 cm (nebulizer) below the umbilicus. Two 12 mm-incisions were made at these localizations (open-laparoscopy) and two 12 mm-balloon trocars (Medtronic®, Autosuture 12 mm, BTT, Covidien) were inserted, ensuring tightness of the abdomen and steadiness of the pressure (Fig. 1). A capnoperitoneum was established and a camera was introduced in the abdomen for a short exploration phase. The nebuliser (MIP®, Reger Medizintechnik, Tottweil, Germany) was connected to the high-pressure injector using a high-pressure injection line (Medrad, Mark 7, Arterion®, Bayer). The distal part of the nebulizer was positioned at a 1 cm depth, as measured from the trocar end. The sheep was placed in Trendelenbourg position to raise the rumen and provide a better exposition of the pelvis. Three milligrams of doxorubicin (Mylan®, 2 mg/mL) diluted in 50 mL saline were nebulized at a flowrate of 30 mL/minute with a maximum pressure of 200 psi, as usually recommended in human patients [19]. After nebulization, the capnoperitoneum was maintained during 30 min. The abdomen was subsequently deflated using an airtight device equipped with a smoke filter and connected to the waste air system in order to avoid contamination of the surgical room with doxorubicin. Thirty additional minutes were allowed for optimum drug penetration in tissues before the animal was euthanized with pentobarbital (Dolethal®, Vetoquinol, 3.6 g, i.e., 20 ml, IV). A median laparotomy was performed and 9 samples (6 peritoneal, 1 ovarian, 1 omental and 1 ceacal) were collected (Fig. 1). One more sample (omentum) was collected just facing the nebulizer. In order to ensure the reproducibility of the sampling for each animal, positions of the peritoneal samples were annotated relatively to their distance to the nebulizer (Fig. 2). Samples were immediately frozen in isopentane at − 40 °C after horizontal inclusion in Optimum Cutting Temperature (Tissue-Tek® O.C.T. Compound, Sakura® Finetek). Blocks were kept frozen at − 80 °C.

### Microscopic analyses

All analyses were performed blindly. The natural fluorescent properties of doxorubicin was used for its localization in the tissues [20]. Samples were handled in a dark room to avoid light exposure that may decrease fluorescence.

Sections (7 μm) were cut using a cryostat (Leica® CM1950), then mounted with 25 μL anti-fade mounting medium (Vectashield®, Vector laboratories) that contained with 4,6-diamidino-2phenylindole (DAPI) at 1/1000. They were kept at 4 °C until observation.

**Fig. 3:**
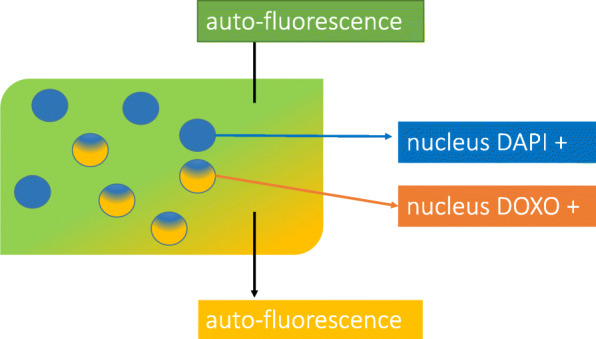
Peritoneum Pictures showing doxorubicin in cell nuclei (nuclei DOXO+ are surrounded with yellow). Doxorubicin is orange color in cell nuclei. DAPI is blue color in cell nuclei. (On the top, blue was cleared to a better visualization or orange)

**Fig. 4:**
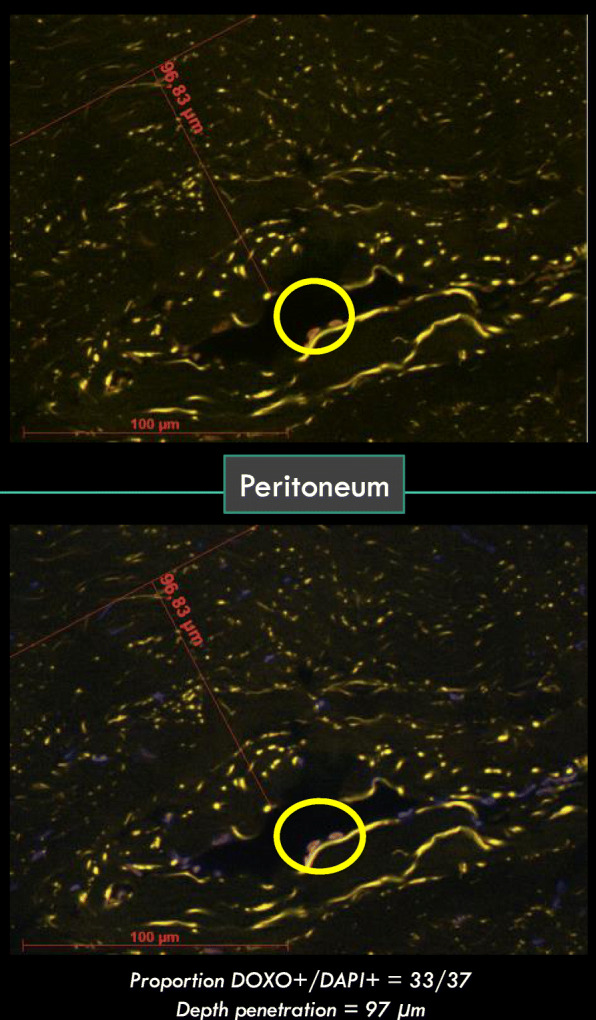
Description and comparison of intra-peritoneal distribution pattern of doxorubicin for each histological type

Analyses were performed with a Carl Zeiss (Germany) AxioObserver Z1 fluorescence microscope equipped with an ApoTome slider and coupled to AxioVision 4.8 software (Zeiss). A complete brightfield view of the section was imaged using a 10x Plan-Neofluar (NA 0.3) objective and 10 square areas of about 200 μm side length were randomly selected. Then fluorescence analysis of each area was performed using a Plan Neofluar X40 oil immersion (NA 1.3) objective and an Axiocam MRm camera (Zeiss). Nuclei were identified using DAPI (blue). Doxorubicin positive nuclei (DOXO+) were stained both in orange and blue. Cytoplasm and extracellular stroma fluoresced in orange together with green auto-fluorescence (Figs. 3 and 4). The time for image acquisition was similar for each fluorochrome throughout the experiments. Fluorescence setup and image acquisition times are detailed in Table 2. Since all images were in the same horizontal plane, fluorescence was not decreased depending on tissue depth.

**Table 2:** Characteristics and signification of fluorescence for orange, blue and green

	Excitation	Emission	Time of acquisition	Origin of fluorescence	Localisation of fluorescence
Vert	470 nm	[500–550]	900 ms	autofluorescence	Extra-nuclear
Bleu	365 nm	> 400	10 ms	**DAPI**^a^	Cell nuclei
Orange	470 nm	> 520	900 ms	Auto-fluorescence	Extra-nuclear
				**DOXORUBICIN**	Cell-nuclei

^a^4,6-diamidino-2phenylindole

### Statistical analyses

Statistical analyses were performed with data collected from the 6 doxorubicin PIPAC-treated sheep. All analyses were performed with SPSS v15.0 and Stata v12.0 software (Stata Corp., College Station, TX, USA). Effect of treatment was analyzed using individual sample location, distance to nebulizer (for peritoneum, distinguishing frontal, proximal and distal samples) and histological type as variables.

Tissue distribution patterns of doxorubicin positive cells were assessed by measuring the ratio of DOXO+/DAPI+ nuclei. For each tissue sample, DAPI + and DOXO + positive cells were counted for each of the 10 square areas and summed up. A Mann-Whitney test was used to analyze the effect of increased intra-peritoneal pressure on the distribution pattern of doxorubicin according to the histological type and location of the sample related to the nebulizer.

Penetration depth of doxorubicin was estimated by measuring the distance between the luminal surface of the tissue and the deepest DOXO+ nuclei that were identified. Samples showing no doxorubicin were removed from analysis. The drug penetration depth was analyzed for each histological type and sample location. Tissue drug penetration was classified in 2 categories: < 100 μm and ≥ 100 μm for group comparison. In order to take into account the correlation between samples from the same ewe, a GEE model (Generalized Estimating Equation) was used [21] to compare penetration depth between the two groups. When one single sample was collected from each animal (ovary, caecum and omentum), drug penetration was compared using a one tailed Chi2 test.

## Results

### Distribution patterns of doxorubicin

No nuclear fluorescence in the > 520 nm wavelength (corresponding to the fluorescence signal emitted by doxorubicin) was observed in any tissue collected in the control ewe.

**Fig. 5:**
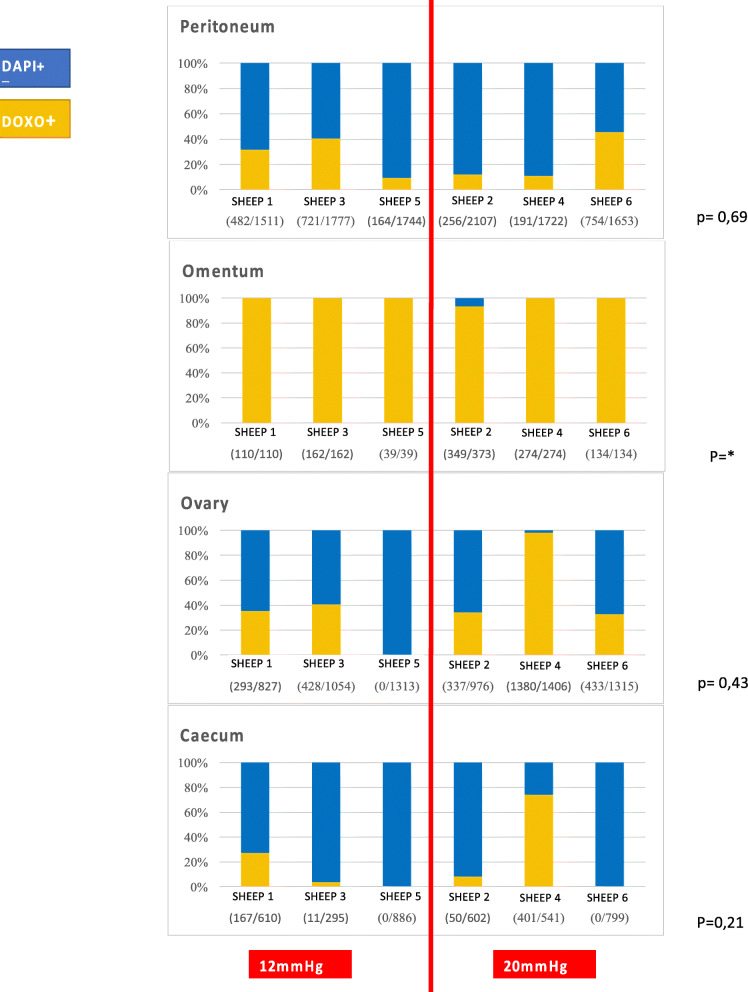
Description and comparison of intra-peritoneal distribution pattern of doxorubicin for each peritoneal localization

**Fig. 6:**
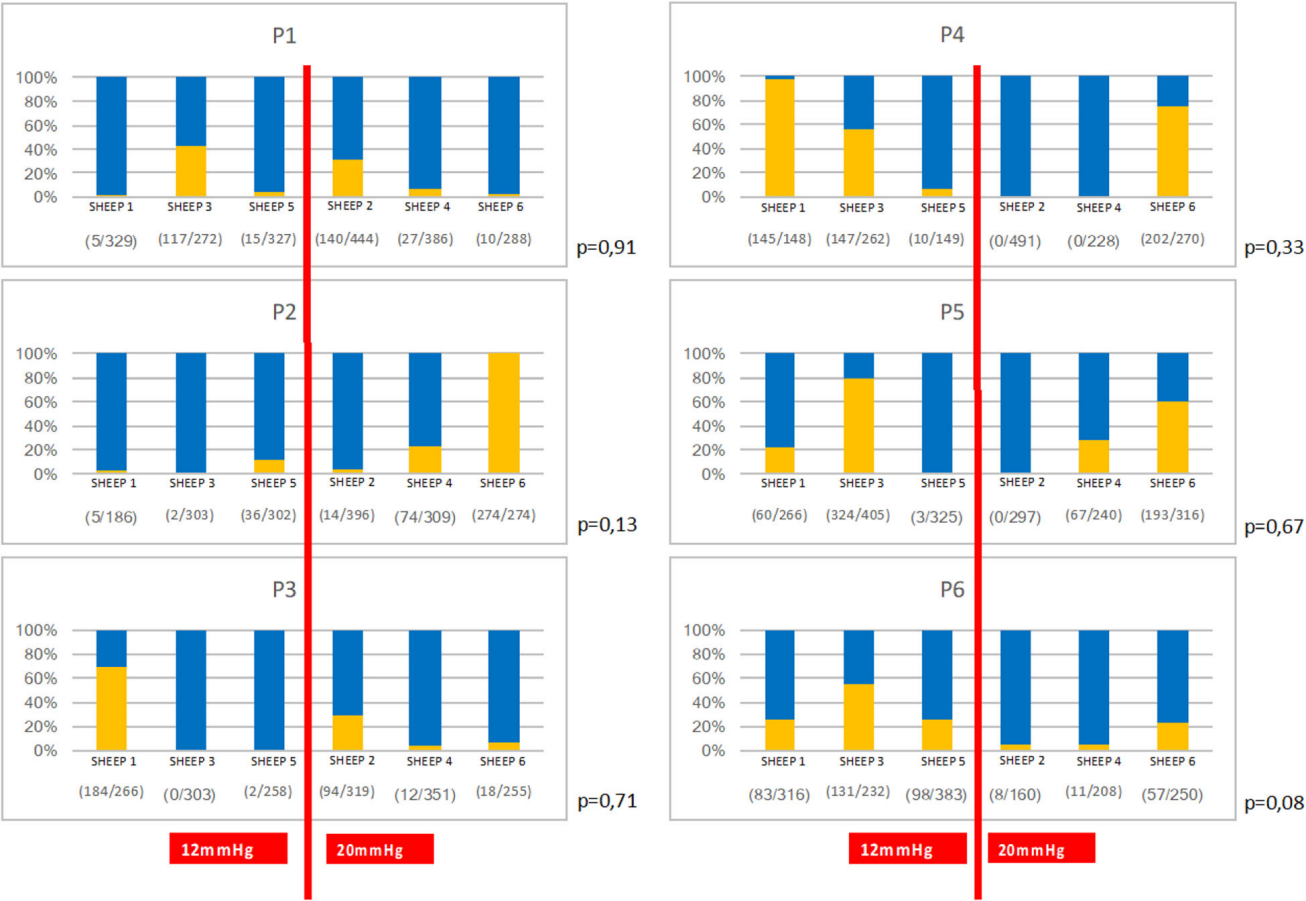
ovary: on the left, no doxorubin is shown. On the right, the other side of the same ovary showed 100% nuclei DOXO+. Pictures showing doxorubicin in cell nuclei. Doxorubicin is orange color in cell nuclei. DAPI is blue color in cell nuclei. (On the top, blue was cleared to a better visualization or orange)

**Fig. 7:**
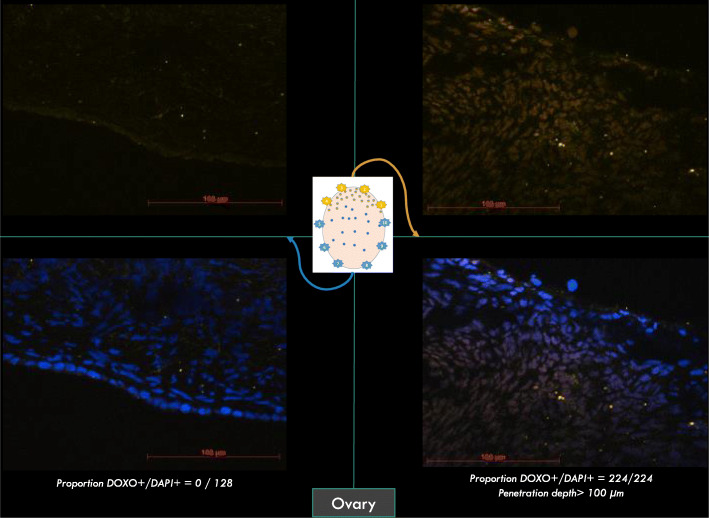
Ovary: on the left, no doxorubin is shown. On the right, the other side of the same ovary showed 100% nuclei DOXO+ Pictures showing doxorubicin in cell nuclei. Doxorubicin is orange color in cell nuclei. DAPI is blue color in cell nuclei. (On the top, blue was cleared to a better visualization or orange)

Doxorubicin was observed in 47 samples of the 54 collected (87%). Pressure increase had no effect on the distribution patterns of doxorubicin regardless of the tissue or peritoneal localization (Figs. 5 and 6). Cell nuclei distribution patterns of doxorubicin were heterogeneous in the peritoneal tissue. Almost all omental nuclei were DOXO+ (99%) whereas the caecum rarely stained positive (17%). Interestingly, in 4 of the 6 ovaries, DOXO+ cells were only found on one side of the ovary and not on the other (Fig. 7).

### Penetration depth of doxorubicin

Similar to cell distribution, penetration depth of doxorubicin was heterogeneous in the peritoneum with no significant difference between groups (*p* = 0,69) when analysed altogether.

Penetration depth was > 100 μm in all group 20 ovarian samples versus only 55% in group 12. There was a significant difference in penetration depth in the caecum between the 2 groups (100% for group 20 versus 22% for group 12). Regarding the omentum, 100% of sampled tissues showed a penetration depth > 100 μm, regardless of the intra-abdominal pressure. These results are summarized in Table 3.

**Table 3:** Comparison of penetration depth of doxorubicin after PIPAC with a pressure at 12 mmHg (group12) and PIPAC with a pressure at 20 mmHg (group20)

Peritoneum	Group 12 (12 mmHg) n/N (%)		Group 20 (20 mmHg) n/N (%)		*p*-value
	34/95	(36)	23/82	(28)	0.69
P1	4/9	(44)	1/16	(6)	0.08
P2	0/11	(0)	10/20	(50)	*
P3	9/10	(90)	3/11	(27)	0.11
P4	16/24	(67)	3/10	(30)	0.38
P5	5/19	(26)	5/17	(29)	0.79
P6	0/22	(0)	1/8	(13)	*
Ovary	**6/11**	**(55)**	**15/15**	**(100)**	*
Omentum	20/20	(100)	26/26	(100)	*
Caecum	**2/9**	**(22)**	**8/8**	**(100)**	*

n = number of samples showing a penetration depth > 100 μm

N = number of samples showing presence of doxorubicin

## Discussion

In this study, a sheep model of PIPAC-doxorubicin was developed to evaluate the impact of intra-peritoneal pressure on two parameters, namely the number of doxorubicin-positive cells and their localization relatively to the surface of the tissue (penetration depth). The sheep is human-sized model, and the same parameters and surgical conditions are used as in human patients, making it very relevant for clinical practice.

This is the first report assessing the impact of increased intra-abdominal pressure on penetration depth of chemotherapy. Penetration depth in the ovaries and caecum was significantly increased with a pressure at 20 mmHg compared to 12 mmHg but this increase was not consistent over all peritoneal samples. In the mouse model, Jacquet and Sugarbaker evaluated the effect of intra-abdominal pressure (12, 20 and 30 mmHg) on doxorubicin concentration in peritoneal tissues after the abdominal cavity was treated with doxorubicin as a simple lavage [22]. They showed that a higher pressure significantly increased doxorubicin penetration into the tissue. Nevertheless, a 30 mmHg intra-abdominal pressure induced toxic effects, especially on digestive organs (necrosis). The impact of increased pressure (5, 10, 15 and 20 mmHg) was also studied in vitro using colon adenocarcinoma cells [23], with cytotoxic effects being significantly increased and proportional to pressure. The same team evaluated the effect of increased pressure on penetration depth of doxorubin in an ex vivo study (fresh porcine peritoneal tissue in a hermetically closed chamber) and did not demonstrate any significant effect [24]. These experiments suggest that peritoneal cells may be less permeable to doxorubicin that other cell types, as also observed in the present study. The formation of a liquid film on the peritoneum after PIPAC may also contribute to the poorer effects of increased intra-abdominal pressure on the peritoneum [25].

Compared to 12 mm HG pressure, intra-abdominal pressure at 20 mmHg did not significantly affect the number of [DOXO+] cells in peritoneal cavity, nor in omemtum, ovary and caecum. Regardless of the pressure, distribution of [DOXO+] cells was heterogeneous and did not reach all areas. These results are consistent with the results of experiments performed in vivo and post mortem on swine [22, 26]. In an attempt to improve these results, electrostatic precipitation of the aerosol (ePIPAC) could help homogenizing the distribution pattern of doxorubicin [27, 28]. Moreover, the omentum, facing the nebulizer, always had the highest number of [DOXO+] cells together with the deepest penetration. In contrast to the omentum, the lowest number of [DOXO+] cells was found in the caecum, which could be explained by anatomical localisation. Apart from the presence of 4 stomachs, the abdominal anatomy of human and sheep is very similar for almost all abdominal organs, but for the bicornuate uterus in sheep instead of uterus simplex in humans. The most voluminous stomach in ruminants is the rumen (anatomically first and largest of the 4 stomachs of ruminants), that occupies the major part of the peritoneal cavity. The caecum was mostly hidden by the rumen during the nebulization process, despite the use of the Trendelenbourg position. This suggests that PIPAC administered chemotherapy does not reach tissues that are positioned beneath other organs, as exemplified with our observations for ovaries where doxorubicin only reached the ovarian side exposed to the nebulization. This observation could have important consequences in clinical practice. Nowadays, patients with recurrent peritoneal carcinomatosis from ovarian cancer often undergo an initial treatment with large abdominal surgery. These surgeries currently induce adherences between organs, thus potentially reducing access to many surfaces at the time when PIPAC is used. In any case, in practice, changing the direction of the trocar during the nebulization may help reach more peritoneal surface.

The data and conclusions drawn from this study deserve to be confirmed with a larger number of animals. Nevertheless, a significant effect of increased intra-abdominal pressure on penetration depth of doxorubicin was observed, suggesting that this should be further explored in clinical conditions. Furthermore, the experiments were performed on healthy tissues and the effect of pressure on doxorubicin penetration could be different on cancerous cells. Peritoneal carcinomatosis is not currently observed in domestic animals and large animal models of peritoneal carcinomatosis are required because laparoscopy and PIPAC could not be performed on rodent nor rabbit models. Finally, it could have been interesting to measure plasma concentration of doxorubicin in the 2 groups in post-operative period to evaluate systemic uptake of doxorubicin. However, sheep were euthanized at the end of procedure as designed in the experimental protocol and this was not performed in the present study.

## Conclusion

Increased pressure was shown to increase penetration depth of doxorubicin in healthy abdominal tissues, suggesting that increased pressure may improve the efficiency of PIPAC on tumoral tissues in clinical practice. In order to confirm these encouraging results, large animal models such as sheep or pigs with peritoneal carcinomatosis should be developed for the benefit of oncologic research and especially PIPAC. Many studies could be performed to evaluate PIPAC efficiency on human patients suffering from carcinomatosis disease. Based on the present data, studies focusing on penetration depth on peritoneal carcinomatosis would be very informative, as these are much more cellular than healthy tissues. It could also be worth comparing the plasma concentrations of chemotherapeutic drugs in relation to variations of the capnoperitoneum.

Moreover, it would be interesting to study whether the efficiency of PIPAC in human patients could be improved from using both ePIPAC and high capnoperitoneum. Finally, the present study could suggest that directing the nebulizer towards the most affected carcinomatosis areas could improve the efficiency of the procedure, which needs to be evaluated.

## Abbreviations

PIPAC: Pressurized Intra-Peritoneal Aerosol Chemotherapy
DOXO +: Doxorubicine fluorescence-positive cell nuclei
DAPI: 4,6-diamidino-2phenylindole
DAPI+: DAPI positive cell nuclei
ENVA: Ecole Nationale Vétérinaire d’Alfort
PC: Peritoneal carcinomatosis
CRBM: Biomedical Research Center
GEE: Generalized Estimating Equation
